# Incomitance and Eye Dominance in Intermittent Exotropia

**DOI:** 10.1167/iovs.17-22155

**Published:** 2017-08

**Authors:** Daniel L. Adams, John R. Economides, Jonathan C. Horton

**Affiliations:** 1 Department of Ophthalmology, University of California, San Francisco, San Francisco, California, United States; 2 Center for Mind/Brain Sciences, The University of Trento, Trento, Italy

**Keywords:** comitance, Hering's Law, strabismus, eye tracking, cover–uncover

## Abstract

**Purpose:**

To determine if the deviation angle changes in subjects with intermittent exotropia as they alternate fixation between the right and left eye in primary gaze.

**Methods:**

In this prospective observational cohort study, 37 subjects with intermittent exotropia were tested for evidence of incomitance. The position of each eye was recorded with a video tracker during fixation on a small central target. A cover–uncover test was performed by occluding one eye with a shutter that passed infrared light, allowing continuous tracking of both eyes. The deviation angle was measured during periods of right eye and left eye fixation. Incomitance was assessed as a function of eye preference, fixation stability, and exotropia variability.

**Results:**

The mean exotropia was 18.2° ± 8.1°. A difference between right exotropia and left exotropia was detectable in 16/37 subjects. Allowing for potential tracking error, the incomitance had a mean amplitude of 1.7°. It was not related to a difference in accommodative effort, eye preference, fixation stability, or variability in deviation.

**Conclusions:**

Comitance is regarded as a feature that distinguishes strabismus from paralytic or restrictive processes. Unexpectedly, eye tracking during the cover–uncover test showed that incomitance is present in approximately 40% of subjects with intermittent exotropia. It averages 10% of the exotropia, and can equal up to 5°. When substantial, it may be worth considering when planning surgical correction.

A deviation of the eyes that remains constant with changes in gaze angle is defined as “comitant.” In comitant strabismus, it has been widely assumed that the eye muscles and their nerves are normal. Misalignment of the eyes is thought to arise from a supranuclear process, such as an imbalance in vergence tone. For example, patients with intermittent exotropia may become symptomatic when their ability to maintain fusion is overcome by excessive divergence or insufficient convergence.^1,2^ Either way, the error in binocular drive is conveyed symmetrically to the ocular motor apparatus, according to Hering's Law. This should produce a comitant deviation, but this prediction has not been verified by precise measurement in a typical cohort of exotropic patients.

Several recent studies have described anatomic abnormalities of the medial rectus muscle in subjects with intermittent exotropia. Magnetic resonance imaging has shown a reduction in muscle volume.^3^ Electron microscopy has revealed atrophy of muscle fibers, with disorganization of sarcomeres and collagen accumulation.^4,5^ The investigators in these studies have suggested that pathologic alterations in the extraocular eye muscles may have a role in the development of intermittent exotropia. This surprising inference opens the door to reconsideration of widely held assumptions about the mechanism of intermittent exotropia. In this spirit, we have assessed comitance in a large population of subjects with intermittent exotropia, by comparing the deviation angle as either eye fixates a central target.

## Methods

Informed consent was obtained from adult participants. Minors granted their assent, and a parent provided informed consent. The study was approved by the University of California, San Francisco (UCSF) Committee on Human Research and by the Kaiser Permanente Northern California Institutional Review Board. It adhered to the tenets of the Declaration of Helsinki.

### Eligibility

All subjects received an ophthalmologic examination that included assessment of the best-corrected visual acuity in each eye, pupils, eye movements, ocular alignment, and stereopsis. Slit-lamp and fundus examinations also were performed. Cycloplegic refraction usually was done at our clinic, although sometimes we accepted data from a referring pediatric ophthalmologist. Eye movement recordings were performed with no refractive correction, except in 3 myopic subjects who wore their contact lenses. Noncontact corrective lens were avoided because they produce reflections that can reduce the accuracy of video eye tracking. After clinical evaluation, eligible patients were scheduled for laboratory testing on a different day.

### Inclusion Criteria

Inclusion criteria were: intermittent exotropia since early childhood, 20/20 Snellen acuity in each eye measured with best correction, no eye or neurological disease except strabismus, no A or V pattern deviation, no dissociated vertical deviation (DVD), absence of diplopia, no pathological nystagmus, intact stereopsis (at least 60 arc-seconds), and no history of ocular surgery.

### Eye Tracker Calibration and Accuracy

**Figure 1:**
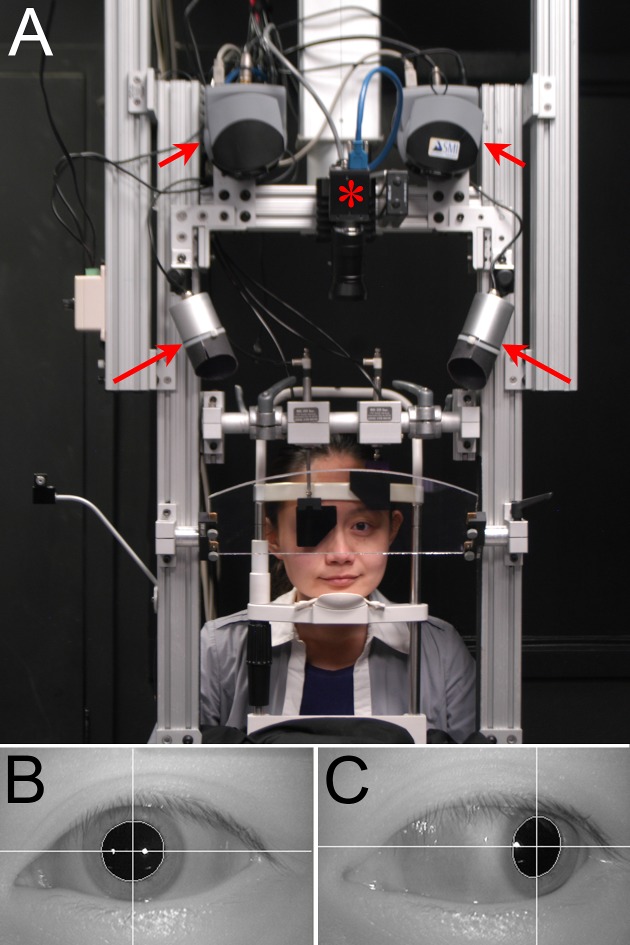
Eye movement recording apparatus. (A) Subject views tangent screen through a hot mirror, with cameras (short arrows) mounted overhead to image each eye. Infrared illuminators (long arrows) are stationed laterally. Infrared filter shutters occlude each eye without interrupting tracking. A central video camera (*) records the experiment. (B) Left eye in primary gaze, with corneal light reflexes from each illuminator. The white crosshair marks the pupil center. (C) Left eye abducted 40°, showing that the temporal light reflex in (B) remains on the cornea, so accurate tracking continues. The nasal light reflex is lost on the conjunctiva.

Eye position was monitored using a separate infrared video camera for each eye (iViewX, SensoMotoric Instruments, Teltow, Germany). The cameras were mounted overhead and a hot mirror was oriented to image the eyes without obstructing the subject's view (Fig. 1A). It is challenging to measure eye position accurately in subjects with a large exotropia, because one eye is deviated far laterally. For video tracking, the limit is reached when the reflex of the infrared illuminator crosses from the cornea to the conjunctiva. To optimize tracking, each camera was situated to image the eye 10° lateral to the eye's line of sight in primary position. More importantly, each infrared illuminator was positioned 30° lateral. Having 2 illuminators generated 2 corneal light reflexes, but the trackers used only the temporal reflex for calculation of gaze angle (Figs. 1B, 1C). It remained on the cornea from 30° medial to 55° lateral, allowing accurate measurement of exotropia over an exceptionally wide range.

Each eye tracker was calibrated by setting the offset and gain independently while the subject fixated on a grid of 9 static points. The other eye was occluded by an infrared filter shutter, positioned to descend by activation of a pneumatic piston. The shutter functioned like an occluder paddle, blocking visible light. However, it passed infrared wavelengths, so eye position was recorded without interruption. For each eye, the calibration was checked by having the subject track a target 0.5° in diameter that moved sinusoidally, first horizontally (±30°) and then vertically (±20°). If inaccurate, an adjustment was made in offset and/or gain, and the calibration was rechecked, during static fixation and sinusoidal tracking.

Video trackers generally are acknowledged to have an accuracy between 0.5° and 1°.^6–9^ The precise value depends on the type of hardware, how it is configured, and physical properties of the eye.^10,11^ In monkeys, a similar system accurately localizes eye position to within 0.1° in primary gaze and 0.6° at 15° eccentric gaze.^12^ Previous measurements have shown an instrument error of ±0.24° in our eye trackers and a standard deviation in eye position of ±0.80° in control subjects fixating a stationary target.^13,14^ To account sufficiently for variation in age, ocular anatomy, and ability to concentrate, we accepted a position accuracy of ±1° for each eye tracker.

### Testing Procedures

Subjects were seated in a dim room, with their head in a conventional chin/forehead rest. Targets were projected onto a tangent screen at a distance of 57 cm, using either a laser or digital projector. For cover–uncover testing, a bright 0.5° spot was projected on the screen center and the subject was asked to fixate assiduously. The occluder was lowered over either eye for 8 seconds and then raised for 10 seconds. The test was repeated 20 to 30 times per eye in most subjects, randomly varying the occluded eye. Eye position was sampled at 120 Hz for off-line analysis.

After cover testing, eye preference was determined using a procedure developed for exotropic monkeys.^14^ Each trial began with a central target. The subject was free to acquire it with either eye. After fixation, the central target was replaced with a peripheral target that appeared at a random location for 200 msec. The subject's task was to saccade to the peripheral target with either eye. The next trial began with presentation of a fresh central target. The percentage of trials initiated by fixation with each eye on the central target provided an index of eye preference. It corresponded consistently to the eye identified as dominant by clinical exam.

### Data Analysis

Traces of eye position from individual trials were overlaid and aligned on pupil occlusion by the infrared shutter. Blinks were excised. Mean traces, with their standard deviations, were generated for each eye. The values for mean position and standard deviation were derived over a variable time window (1–4 seconds), starting after the deviated eye reached a steady position. This time window was equal for the deviating and fixating eyes for any given condition, but could vary between conditions (i.e., left versus right exotropia) or subjects. It was tailored so that measurements were made over the maximum time available. The mean exotropia during right versus left eye fixation was compared using the Wilcoxon rank sum test.

## Results

The 37 subjects with intermittent exotropia (16 male, 21 female) who contributed to this study had a median age of 30 (range, 7–65).

### Incomitance in Intermittent Exotropia

**Figure 2:**
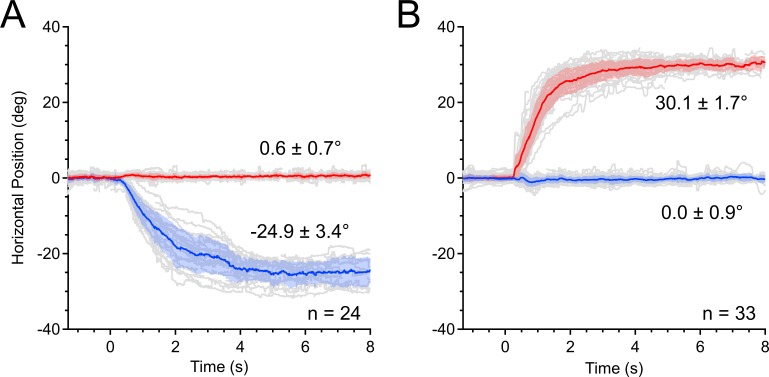
Incomitance in exotropia. Recordings from an 11-year-old girl with a right eye fixation preference and a refraction of: −2.25 + 2.50 × 98 (right eye) and −4.25 + 3.75 × 80 (left eye). At t = 0 seconds, a shutter covered either eye, causing it to move outwards. Mean position is shown for the right (red) and left (blue) eye, with shading denoting standard deviation. Positive values represent right gaze. n = number of trials. (A) Shutter occlusion of the left eye produced an exotropia of 25.5° (|−24.9° – 0.6°|). The fixating right eye's mean position deviates from 0° by less than tracking error. (B) Shutter occlusion of the right eye produced a deviation of 30.1°.

Figure 2 shows eye movement recordings from an 11-year-old girl with intermittent exotropia who had an incomitance of 4.6°. When deviated, she could alternate fixation on visual targets, but spontaneous exotropia occurred only in the left eye. On cover testing her left exotropia was 25.5° (Fig. 2A). Her right exotropia was larger, measuring 30.1° (Fig. 2B). The variability in position of the deviated eye also differed, for left versus right. The position of the exotropic left eye had a standard deviation of 3.4°, whereas that of the exotropic right eye was only 1.7°.

**Figure 3:**
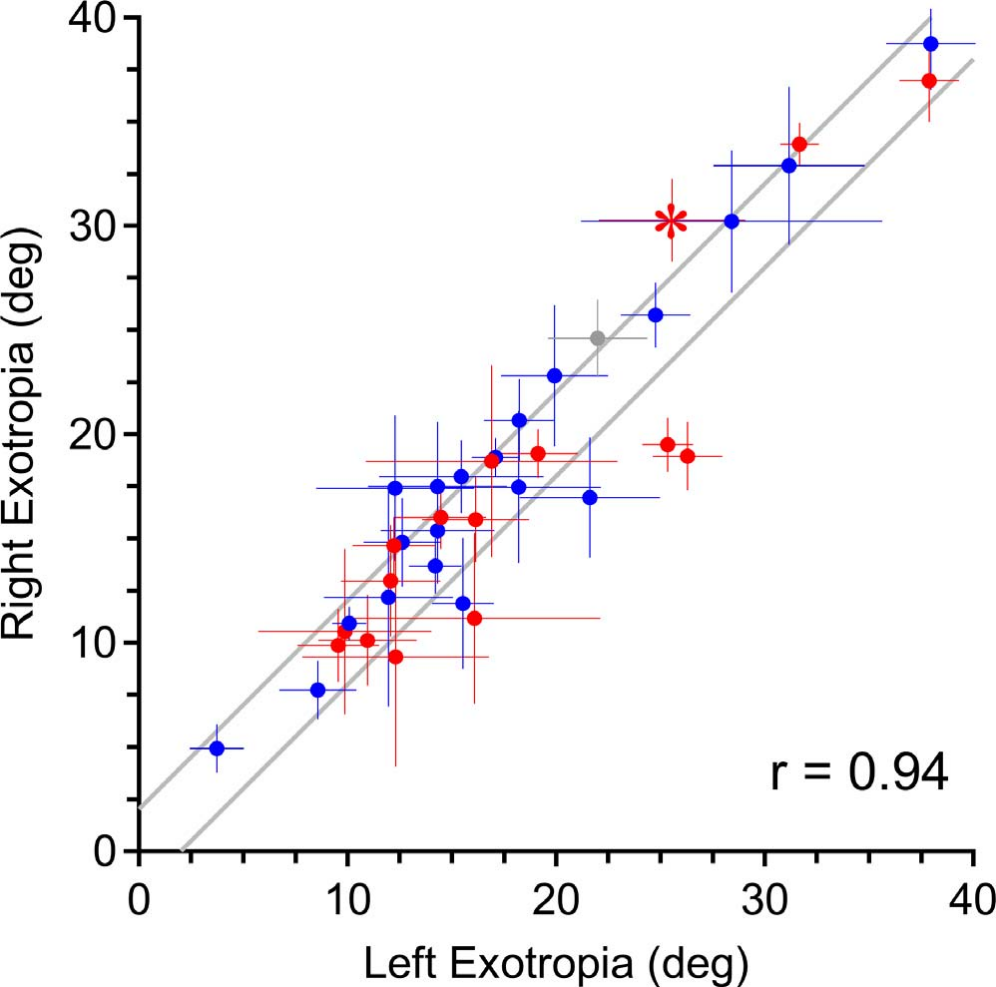
Scatter plot showing a correlation between left and right exotropia (r = 0.94) for 37 subjects, measured with the fixating eye in primary gaze and the deviated eye occluded. The gray lines define the maximum deviation from the unity line attributable to tracking error (±1° for each eye tracker). 16/37 subjects fall outside these error bounds, indicating the presence of incomitance. Horizontal bar: SD of left exotropia. Vertical bar: SD of right exotropia. Blue = left eye dominant, red = right eye dominant, gray = no eye dominance. *Patient illustrated in Figure 2.

Intermittent exotropia generally is regarded as a comitant disorder. Nonetheless, it was not uncommon to detect a difference between right and left exotropia. To address this point, strabismic deviation was plotted versus eye of fixation for all subjects (Fig. 3). The mean exotropia was 18.2° ± 8.1°. The median exotropia was 16.0° (Q_25_ – Q_75_ = 13.4° – 22.4°). The scatterplot showed that the magnitude of right and left exotropia was highly correlated (*r* = 0.94, Pearson correlation). However, some subjects showed a small discrepancy in exotropia, depending on the eye of fixation. Given potential tracker position error of ±1°, only an incomitance exceeding 2° was deemed meaningful. Among the 37 subjects, 16 (43%) met this threshold. For each of these 16 patients, the incomitance was significant (*P* = 0.001), averaging 3.7° ± 1.5° and ranging up to 7.3°. Given that up to 2° of the difference might be explained by tracking error, the incomitance averaged at least 1.7°. There was no trend for a larger exotropia to be correlated with a greater amount of incomitance. This might be evident if our findings were an artifact, caused by inaccurate measurement of large strabismus angles.

A difference in refractive error between the eyes could explain the incomitance present in some subjects with exotropia. A switch in fixation to an eye with a more hyperopic error would result in increased accommodative demand and, hence, reduced exotropia angle. For example, the patient illustrated in Figure 2 had a spherical equivalent of −1.00 in the right eye and −2.375 in the left eye. To focus on a target at 57 cm (−1.75 diopters) required 0.75 diopters accommodation in the right eye and none in the left eye. An incomitance of 4.6° generated by a 0.75 diopter difference in accommodative effort would correspond to an accommodative convergence/accommodation (AC/A) ratio of 6.1°/diopter (11 prism-diopters/diopter). This is 3 standard deviations greater than the average AC/A ratio, which equals 3.5 prism-diopters/diopter.^15,16^ Therefore, in this patient, asymmetrical accommodative effort contributed to incomitance, but was unlikely to account for it fully.

**Figure 4:**
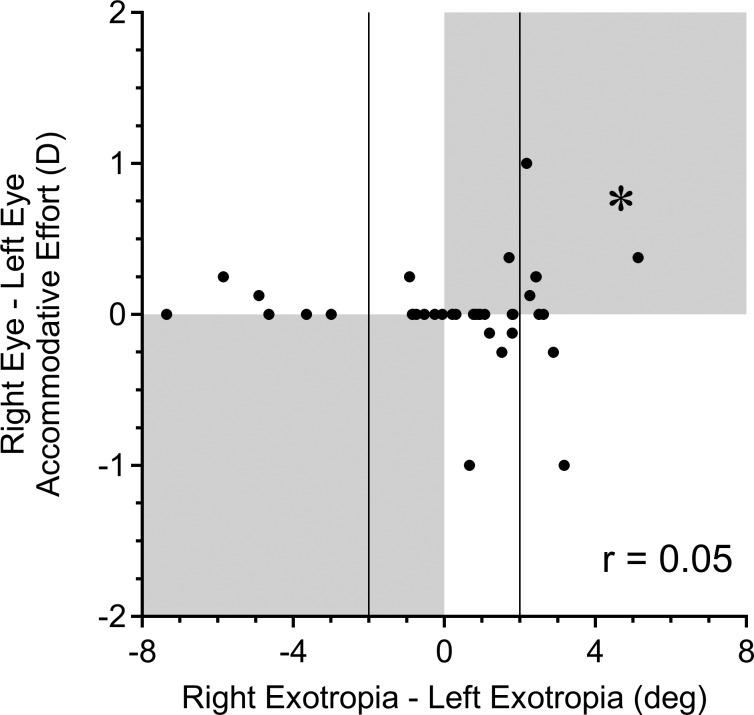
Difference in accommodative effort (D = diopters exerted by each eye) required to focus at 57 cm versus incomitance, with the fixating eye in primary gaze. The same accommodative effort was required in each eye in the majority of subjects, accounting for the clustering of points along the horizontal meridian. By definition, incomitance is present only in subjects with more than 2° difference in exotropia (vertical lines). Accommodative effort could contribute to incomitance only for subjects whose data fall in shaded quadrants. A difference in accommodative effort of >0.25 diopters potentially contributed to incomitance >2° in only 3/16 patients. *Patient illustrated in Figure 2.

To address this issue systematically, the accommodative effort required by each eye to focus on a target at 57 cm was calculated (Supplementary Table). No accommodative effort was required for any eye more myopic than −1.75 diopters (1.00/0.57). A plot of difference in accommodative effort between the eyes versus amount of incomitance (Fig. 4) showed no correlation (*r* = 0.05). In fact, only 4/16 patients with detectable incomitance had an interocular difference in accommodative effort exceeding 0.25 diopters. Only 3 of these 4 patients displayed a smaller exotropia when fixating with the eye that required a greater accommodative effort.

### Eye Dominance in Intermittent Exotropia

Of the 16 incomitant subjects, all but one had a clearly dominant eye, evinced by capture of the central fixation target with that eye on >75% of trials. Among the 8 left eye dominant subjects, 6 had a larger right exotropia. Among the 7 right eye dominant subjects, 4 had a larger left exotropia. Therefore, in 10/15 subjects, exotropia was larger when the dominant eye was engaged in fixation. The probability of 10 or more subjects exhibiting this property by chance is 0.15. Therefore, our data showed no relationship between incomitance and eye dominance. A larger population of patients would need to be tested to exclude the possibility that exotropia is larger when the dominant eye fixates.

**Figure 5:**
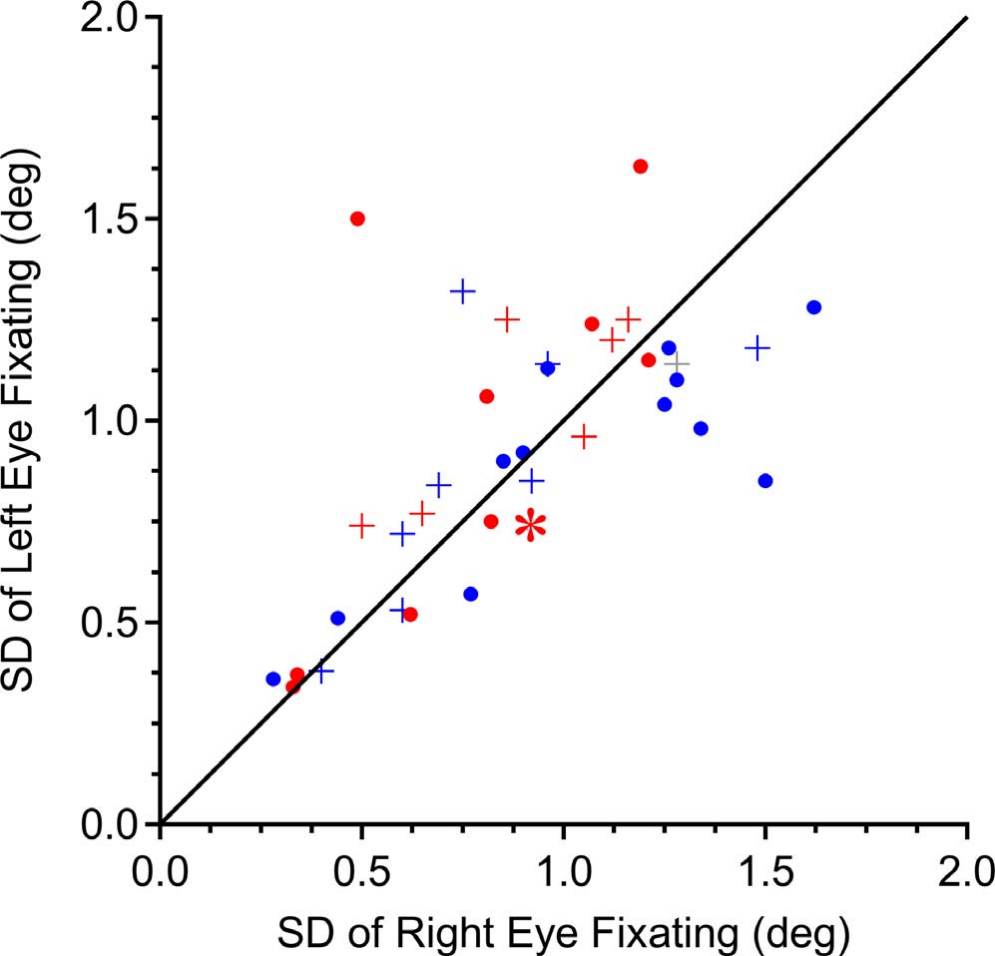
Eye preference is not correlated with stability of the fixating eye. Data were collected while one eye was fixating and the other was occluded. The standard deviation of the position of the fixating right eye versus the fixating left eye was correlated for individual strabismic subjects (r = 0.66). However, eye dominance (red = right eye; blue = left eye) did not confer better fixation stability. If it did, red points would lie above the unity line, and blue points would fall below. +, subjects with > 2° incomitance. *Patient illustrated in Figure 2.

It is unclear what factors give rise to eye dominance in intermittent exotropia, in the absence of amblyopia or refractive error difference. In subjects with exotropia, fixation stability is impaired. The eye engaged in fixation is slightly less stable in position than the fixating eye of normal, binocular subjects.^13^ It is possible that exotropic subjects prefer a given eye simply because it is able to maintain steadier fixation upon a visual target. Figure 5 compares the standard deviation of each eye's position during fixation. The mean standard deviation in position was 0.9°. There was a correlation between the stability of the right and left eyes for individual subjects (*r* = 0.66). If fixation stability were better in the dominant eye, points representing right dominant patients (red) would be situated above the unity line and those belonging to left dominant patients (blue) would lie below. Instead, red and blue points were scattered on both sides of the line. This was true even when only the 16 patients with significant incomitance were considered.

**Figure 6:**
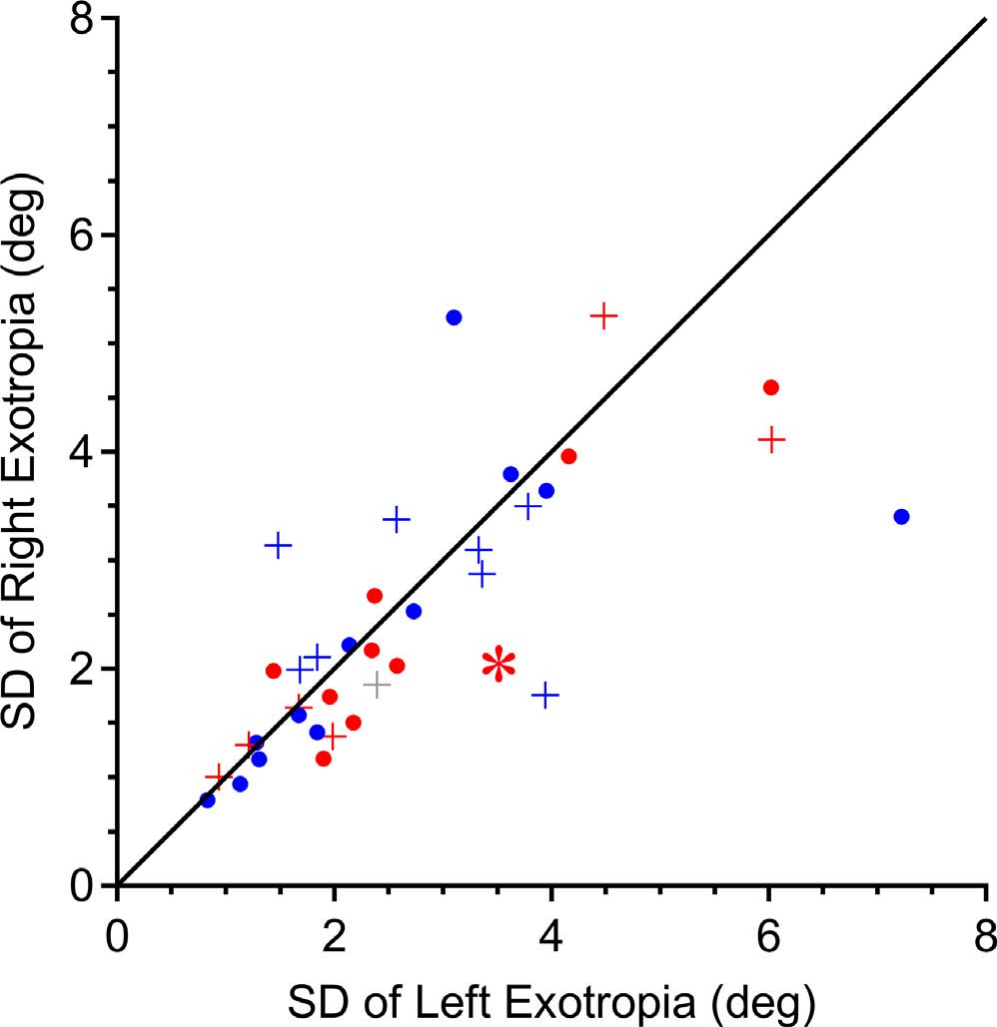
Eye preference is not correlated with variability of deviation angle. The standard deviation of exotropia is plotted for the right eye fixating and for the left eye fixating for each subject. The standard deviations are correlated (r = 0.74), but there is no clear relationship with eye dominance (red = right eye dominant, blue = left eye dominant). If fixation by the dominant eye rendered the exotropia more stable, blue points would be located above the line and red points below. +, subjects with > 2° incomitance. *Patient illustrated in Figure 2.

Another explanation for eye dominance might be that it conveys a more stable angle of ocular misalignment. Figure 6 plots the standard deviation of each subject's left versus right exotropia, coded for eye dominance. Individuals varied widely in the amount by which their exotropia fluctuated. This property largely was due to instability in the position of the covered, deviated eye.^13^ The mean standard deviation of exotropia amplitude was 2.6°. For any given subject, the standard deviations of the left and right exotropia were well correlated (*r* = 0.74). This similarity suggested that exotropia variability is not related to eye dominance. In fact, right dominant patients showed no tendency for their left exotropia to be less variable than their right exotropia. By the same token, left dominant patients did not have a less variable right than left exotropia.

## Discussion

The patients we tested were similar to populations described in previous studies and, therefore, were likely representative of typical patients with intermittent exotropia.^1,17–25^

The main finding was that nearly half the subjects demonstrated incomitance. For these patients, the average discrepancy in deviation angle was 3.7°. This figure includes a potential position error of up to 1° generated by each eye tracker. Given the prevailing belief that exotropia is comitant, the most conservative stance is to assume that tracking error in each eye contributed maximally to the apparent incomitance. Subtracting each tracker's error yields a mean incomitance of only 1.7°. This was approximately 10% of the mean exotropia demonstrated by incomitant patients. It was a modest degree of incomitance, which could be missed even by a careful examiner performing a cover–uncover test using hand-held prisms. In boxed sets, prism power changes in 5 prism-diopter steps for values ≥15 prism-diopters. Consequently, the magnitude of exotropia often is not measured precisely. Moment-to-moment variability in the angle of exotropia adds to measurement uncertainty in the clinical setting.^13,19^

A difference between the eyes in accommodative effort would be a trivial explanation for incomitance.^26^ However, most patients in our cohort had no difference in refractive error between the eyes. Unequal accommodation was a potential factor in only 3/16 subjects with incomitance (Fig. 4).

We did not measure accommodative effort, but inferred it from each subject's refractive error. It is possible that some subjects did not make a full effort to focus each eye on the fixation target. However, Yang and Hwang^27^ have shown that subjects with intermittent exotropia display only a 0.20 diopter asymmetry in accommodative response between the dominant and nondominant eye during monocular fixation of near targets. This modest difference, and the lack of any relationship between eye dominance and incomitance (Fig. 3), supports our view that unequal accommodation was not responsible for incomitance. We also found that eye dominance showed no consistent relationship to stability of fixation (Fig. 5) or stability of exotropia (Fig. 6).

Dissociated horizontal deviation (DHD) is a special form of unequal exotropia that occurs not from a shift in gaze angle, but from a change in eye of fixation.^28^ Romero-Apis and Castellanos-Bracamontes^29^ reported that DHD was present in 17/565 patients with exotropia. All 17 patients also had DVD. We monitored vertical eye position closely, and excluded all subjects with DVD because their exotropia may be atypical. We also excluded patients with a history of eye muscle surgery, because incomitance might be a postoperative artifact.

DHD can be distinguished from horizontal incomitance by performing the reversed fixation test.^30–32^ In a laboratory setting, the ideal paradigm would present a different fixation target to each eye, separated by the subject's deviation angle. For an exotrope, each eye's target would be located on the corresponding side of the screen. As the subject alternated fixation on each target, the eyes would hardly move, so any change in deviation angle would represent DHD. Unfortunately, as our subjects performed a cover–uncover test, they changed gaze angle and eye of fixation simultaneously. Therefore, we cannot be sure if the difference we measured in deviation angle in 16/37 subjects was due to DHD or incomitance. We favor incomitance, but our testing did not exclude the possibility of DHD.

In our subjects, deviation angle was measured while one eye was occluded. It is possible that the incomitance recorded in some subjects might not be present during binocular viewing. The impact of monocular occlusion in exotropia has been tested by many different investigators, sometimes with conflicting results.^33–37^ In every study, occlusion was performed for a prolonged period, ranging from half an hour to days. Recently, it has been shown that the deviation angle measured during a brief cover test matches the amplitude of exotropia present with both eyes open, with few exceptions.^38^ Therefore, it seems doubtful that incomitance was an artifact of making measurements with one eye occluded, and indeed, the cover–uncover test is used routinely in the clinic to measure exotropia magnitude.

Our measurements of exotropia were made at near rather than far. The deviation angle at near sometimes is less than the angle at distance, but the impact on incomitance is unknown.^39^ It would be worthwhile to repeat our experiments with subjects fixating on a far target, to determine if incomitance also is present.

Our finding of incomitance in intermittent exotropia is not wholly unexpected, in light of previous reports describing lateral incomitance. Moore^40^ was first to recognize variation in exodeviation amplitude with changes in gaze angle. She found reduced exotropia on full lateral gaze, perhaps owing to asymmetric force exerted by a relaxed but stretched medial rectus muscle.^41^ Repka and Arnoldi^42^ demonstrated that lateral incomitance can occur from measurement error, if neutralizing prisms are rotated improperly. However, after correcting for this artifact, 9% of their patients still had lateral incomitance. In our testing apparatus, patients shifted their gaze angle by alternating fixation on a central target. Although they did not move their eyes into full lateral gaze, a fraction of the incomitance evoked by such a maneuver would be expected from the smaller shifts in gaze angle that they did make.

Lateral incomitance can be large enough to influence surgical planning.^41,43^ In a recent study, 63/155 patients (41%) undergoing surgery for intermittent exotropia had lateral incomitance of >5 prism-diopters.^44^ For patients with sizeable incomitance in primary gaze, such as those identified in our study, one might consider performing slightly asymmetrical surgery to obtain optimal results. For example, in a patient with a right larger than left exotropia, one might recess the right more than the left lateral rectus.

The genesis of the small incomitance that can occur in intermittent exotropia remains obscure, as does the basic mechanism of the disease itself. Ultimately, the explanation for intermittent exotropia will have to account for the fact that in some subjects, it is not a perfectly comitant disturbance of gaze.

## Supplementary Material

Supplement 1
