# Supplementary material for: Incomitance and Eye Dominance in Intermittent Exotropia
Source: Invest Ophthalmol Vis Sci. 2017 Aug;58(10):4049–55. doi: 10.1167/iovs.17-22155 (PMC5559177; doi:10.1167/iovs.17-22155)
Supplement: Supplement 1 [file iovs-58-07-58_s01.pdf]

Supplementary Table 1:
Accommodative Effort and Incomitance

			OD		OS			
subject	sex	age	spherical equivalent (diopters)	accommodative effort at 57cm (diopters)	spherical equivalent (diopters)	accommodative effort at 57 cm (diopters)	OD - OS accommodative effort (diopters)	R XT - L XT (degrees)
1	m	12	0	1.75	0	1.75	0	-0.9
*2	m	43	-7.875	1.75	-7.75	1.75	0	-0.7
3	m	31	-3.25	0	-3	0	0	-0.5
4	m	35	-0.875	0.875	-1.25	0.5	0.375	1.7
5	f	55	0	1.75	0	1.75	0	1.8
6	f	7	-1.25	0.5	-1.25	0.5	0	0.3
7	f	26	1	2.75	2	3.75	-1	0.7
8	m	65	-1.5	0.25	-1.625	0.125	0.125	2.3
9	m	8	-0.25	1.5	-0.25	1.5	0	0.2
*10	f	15	-5.5	1.75	-4.875	1.75	0	1.1
11	f	12	-0.5	1.25	-1.5	0.25	1	2.2
12	m	16	-0.125	1.625	-0.375	1.375	0.25	-5.9
13	f	52	-4.375	0	-2.75	0	0	-0.3
14	f	61	-1.5	0.25	-1.5	0.25	0	2.5
15	f	21	-0.25	1.5	-0.5	1.25	0.25	2.4
16	m	61	-4.25	0	-4.5	0	0	-7.3
17	f	51	3.875	5.625	3.5	5.25	0.375	5.1
18	f	15	-1	0.75	-0.875	0.875	-0.125	1.8
19	f	30	0	1.75	0	1.75	0	-3.0
20	f	22	-0.75	1	-0.75	1	0	-4.6
21	m	32	-0.25	1.5	-0.25	1.5	0	0.9
22	m	52	-2.875	0	-0.75	1	-1	3.2
23	f	22	0	1.75	-0.25	1.5	0.25	2.4
24	m	15	-1.75	0	-1.5	0.25	-0.25	2.9
25	m	46	0	1.75	0	1.75	0	0.9
*26	f	36	-7.875	1.75	-8.5	1.75	0	1.8
27	f	35	-1.125	0.625	-1	0.75	-0.125	1.2
28	f	28	-5	0	-5.25	0	0	0.8
29	m	47	0.125	1.875	-0.125	1.625	0.25	-0.9
30	f	12	-1.75	0	-2	0	0	2.6
31	m	12	0.75	2.5	0.625	2.375	0.125	-4.9
32	f	31	0	1.75	0	1.75	0	-3.7
33	f	36	-1	0.75	-1	0.75	0	-0.1
34	f	9	-1.75	0	-1.5	0.25	-0.25	1.5
35	f	11	-1	0.75	-2.375	0	0.75	4.7
36	m	40	-5.25	0	-1.75	0	0	0.8
37	m	11	0	1.75	0	1.75	0	-0.8

Supplementary Table 1:

- Spherical equivalent was determined by refraction under cycloplegia. Retinoscopy was performed, followed by subjective refraction. Subjects were tested without correction, except 3 denoted by * wore contact lenses.
- To focus on a spot at 57 cm, an accommodative effort of 1.75 diopters is required for a subject with a plano refraction. No accommodative effort is required for a subject with a refraction of $\geq$ -1.75 diopters.
- 21/37 subjects showed no difference in accommodative effort between the eyes.
- 6/37 subjects showed more than 0.25 diopters difference in accommodative effort.
- Of these 6 subjects, only 4 showed measurable (> 2°) incomitance.
- Of the 4 subjects with $\geq$ 0.25 diopters difference in accommodative effort and measurable incomitance, only 3 subjects (11, 17, 35) show a potential effect, i.e., a greater accommodative effort in the fixating eye accompanied by a smaller exotropia in the other eye.
